# Growth Properties of Carbon Nanowalls on Nickel and Titanium Interlayers

**DOI:** 10.3390/molecules27020406

**Published:** 2022-01-09

**Authors:** May Tran Thi, Seokhun Kwon, Hyunil Kang, Jung-Hyun Kim, Yong-Kyu Yoon, Wonseok Choi

**Affiliations:** 1 Department of Electrical Engineering, Hanbat National University, Daejeon 34158, Korea; tranthimayk61hoa@gmail.com (M.T.T.); kwon1567@naver.com (S.K.); hikang@hanbat.ac.kr (H.K.); 2 Department of Advanced Materials Engineering, Hanbat National University, Daejeon 34158, Korea; jhkim2011@hanbat.ac.kr; 3 School of Electrical and Computer Engineering, University of Florida, Gainesville, FL 32611, USA; ykyoon@ece.ufl.edu

**Keywords:** carbon nanowall, RF-magnetron sputtering, PECVD, interlayer, thickness

## Abstract

This research is conducted in order to investigate the structural and electrical characteristics of carbon nanowalls (CNWs) according to the sputtering time of interlayers. The thin films were deposited through RF magnetron sputtering with a 4-inch target (Ni and Ti) on the glass substrates, and the growth times of the deposition were 5, 10, and 30 min. Then, a microwave plasma-enhanced chemical vapor deposition (PECVD) system was used to grow CNWs on the interlayer-coated glass substrates by using a mixture of H_2_ and CH_4_ gases. The FE-SEM analysis of the cross-sectional and planar images confirmed that the thickness of interlayers linearly increased according to the deposition time. Furthermore, CNWs grown on the Ni interlayer were taller and denser than those grown on the Ti interlayer. Hall measurement applied to measure sheet resistance and conductivity confirmed that the electrical efficiency improved significantly as the Ni or Ti interlayers were used. Additionally, UV-Vis spectroscopy was also used to analyze the variations in light transmittance; CNWs synthesized on Ni-coated glass have lower average transmittance than those synthesized on Ti-coated glass. Based on this experiment, it was found that the direct growth of CNW was possible on the metal layer and the CNWs synthesized on Ni interlayers showed outstanding structural and electrical characterizations than the remaining interlayer type.

## 1. Introduction

Carbon-based materials are easy to process, relatively inexpensive, chemically and structurally stable, and have high electrical conductivity [1,2,3,4]. For these reasons, they have attracted attention for a long time, and many studies on their application on semiconductors, sensors, thin films, materials, storage, and displays have been conducted [5,6,7]. Among carbon-based nanomaterials, the carbon nanowall (CNW), which was discovered while synthesizing carbon nanotubes (CNTs), is a type of material wherein multilayers of graphene are vertically synthesized on a circuit board [3,4]. Moreover, the CNW has the widest reactive surface, as well the as merits of excellent electron mobility, mechanical strength, and chemical stability, due to its carbon and graphene components. The physical properties and the shape of CNWs are highly dependent on the processing conditions and the quality of the substrates used [1,2,3,4,5,6,7]. In addition, the electrical characteristic of CNWs is changed by parameters, such as area density, height, and thickness. 

However, CNWs are difficult to deposit on a glass substrate due to their low adherence. Under very small stresses, the walls easily break down, and the substrate and CNW can be separated [8,9,10,11]. In order to solve these issues and increase the utilization of CNW for devices, an intermediate layer (interlayer) was inserted between the CNW and the substrate. Recently, titanium is a widely used interlayer with excellent thermal stability and chemical safety, as well as a high fine hardness that can dissolve the oxides remaining on the substrate surface and act as a compliant layer that reduces shear stress at the coating–substrate interface [12,13]. Meanwhile, the nickel interlayer provides excellent adhesion properties for the subsequent coating layers [14]. The unique functional properties of nickel include its ability to be slowly oxidized by air at room temperature, as well as its corrosion resistance, hardness, and erosion resistance, and uniform layer thickness, even on complicated components [15].

In this work, nickel (Ni) and titanium (Ti) are used as intermediate layers, then the CNW is grown by injecting a mixture of hydrogen (H_2_) and methane (CH_4_) gases into the interlayer deposited in a microwave plasma-enhanced chemical vapor deposition (PECVD) chamber. The interlayer features are evaluated, and the growth characteristics of CNW grown directly on the glass substrate are compared to those of the CNW grown on the interlayer.

## 2. Experimental Details

### 2.1. Interlayer Deposition

The 2 × 2 cm^2^ glass substrates were cleaned with an ultrasonic cleaner using trichloroethylene (TCE), acetone, methanol, and deionized (D.I.) water, in that order, for 10 min each, to remove impurities except for the gases required to synthesize the CNWs. Nitrogen gas was used to dry the cleaned glass. The cleaned glass wafers were then placed in a radio frequency (RF) magnetron sputtering system chamber with a base vacuum maintained below 10^−5^ Torr. Ar gas (40 sccm) was injected as the sputtering gas, and the working pressure was maintained at 3 × 10^−3^ Torr. Four-inch Ni and Ti targets were used. The deposition time (5, 10, and 30 min) was varied at 50 W RF power and 1.0 × 10^−2^ Torr working vacuum, and each was attached to a substrate via spinning at 1700 rph during deposition.

### 2.2. Carbon Nanowall Growth

For the synthesis of the CNWs, microwave PECVD was used. A base pressure of 1 × 10^−5^ Torr was maintained in the PECVD chamber, methane CH_4_ gas was injected into the chamber at 20 sccm, and H_2_ gas was injected at 40 sccm. In this stage, the working pressure in the chamber was kept at 1 × 10^−2^ Torr, and the CNWs were synthesized by using 1200 W microwave power at 500 °C for 5 min. After the growth was completed, the wafer was slowly cooled to room temperature, and then the CNWs grown on interlayer-coated glass substrates were taken out from the chamber. The detailed experiment conditions are summarized in Table 1 and Table 2.

### 2.3. Analysis and Measurement

We used field emission scanning electron microscopy (FE-SEM, Hitachi S-4800, Krefeld, Germany) to analyze the types and properties of the CNWs on interlayer-coated glass substrates. In addition, Hall measurement system (Ecopia, HMS-3000, Toronto, ON, Canada) and UV-Vis equipment (SCINCO, S-3100, Seoul, Korea) were used to verify the electrical characteristics of grown CNWs and their light transmittance, respectively.

## 3. Results and Discussion 

### Surface and Structural Characteristics

**Figure 1:**
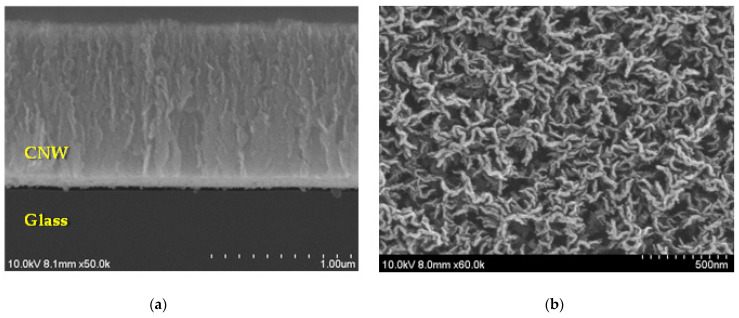
FE-SEM images of the grown CNW on the glass substrate: (**a**) cross-sectional image and (**b**) surficial image.

Figure 1 shows the surficial and cross-sectional SEM images of the synthesized CNWs directly on glass substrates. As shown in the figure, the CNWs were grown into uniform shapes and to heights of about 805 nm. However, the cross-sessional and planar FE-SE images of the CNWs reveal a gap between the two layers of material. In particular, the dark grey color is the glass substrate, the thin black layer is the gap and a thin intermediate layer is the carbon layer. This phenomenon is thought to be caused by the low adherence to the glass substrate of the CNWs [16] as well as the influence of low growth temperature.

**Figure 2:**
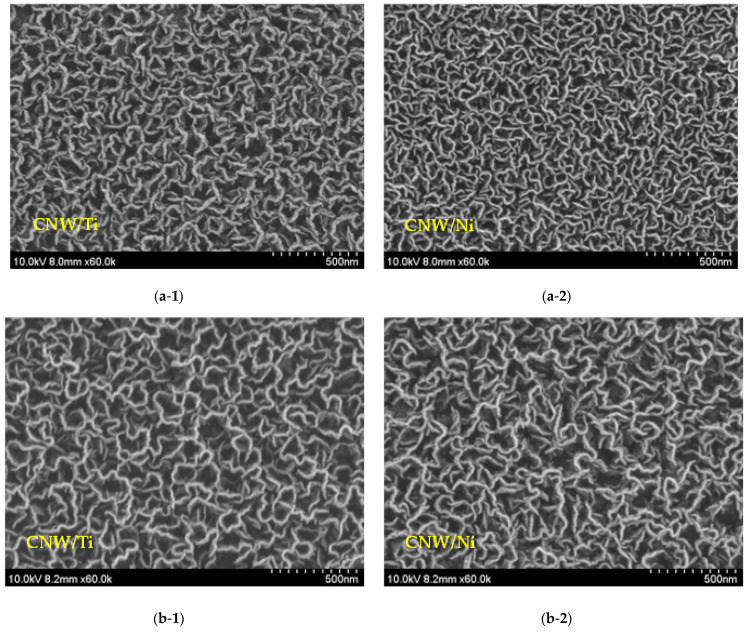

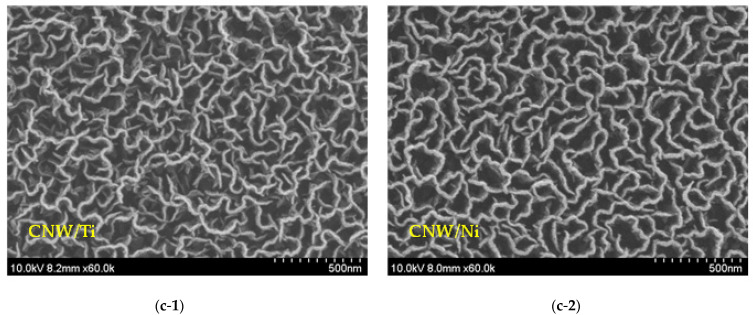
Surface SEM images of the grown CNWs depending on the different interlayers and deposition time: (**a**-**1**,**a**-**2**): 5 min; (**b**-**1**,**b**-**2**): 10 min; and (**c**-**1**,**c**-**2**): 30 min.

Figure 2 shows the surface FE-SEM images of the grown CNWs after Ni or Ti were deposited on the glass substrates. In both types of interlayers, the basis of the CNW shape was established better than in the CNW grown directly on the substrate. According to many studies that have been performed, the 10 nm Ni metal layer acted as a catalyst, and CNT was grown instead of CNWs [17]. However, all interlayers in this study were created with thicknesses larger than 10 nm, allowing the CNWs to develop successfully on stable substrates. For both types of the inserted interlayer on substrates, each of the synthesized CNWs formed a thin monolayer with a unique maze-like structure, and the two-dimensional carbon seats were well grown vertically on the interlayers. 

Furthermore, when comparing the density of a CNW synthesized on the Ni and Ti interlayers, the density of the CNW synthesized on the Ni interlayer was higher than the density of the CNW synthesized on the Ti interlayer. This may be due to the difference in thermal conductivity of nickel and titanium on glass substrates as well as the fact that Ti oxidizes to TiO_2_ when exposed to the atmosphere, reducing the uniformity of the substrate utilized for CNW development. The change in the CNW shapes can also be identified based on the sputtering duration (5, 10, and 30 min). When the deposition period was extended, the form and density of CNWs on Ti-interlayer-coated glass substrates were essentially the same; however, there was a substantial variation on the Ni-interlayer-coated glass substrates.

**Figure 3:**
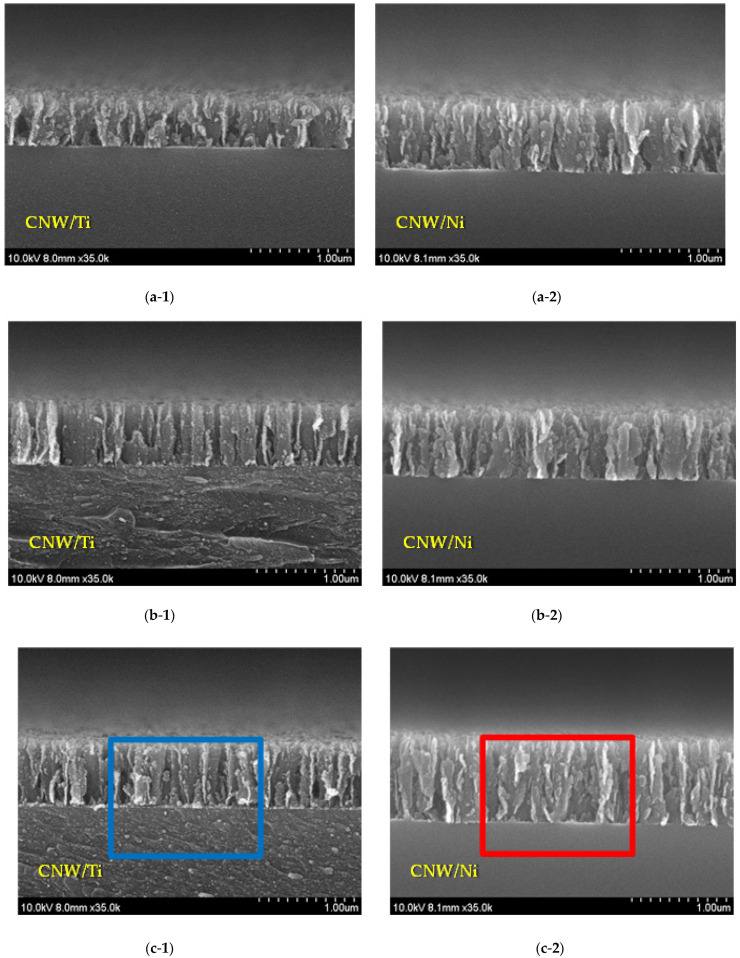

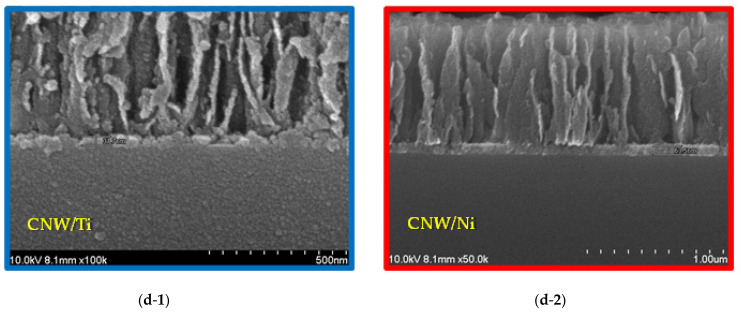
Cross-sectional SEM images of the CNWs depending on the different interlayers and their deposition time: (**a**-**1**,**a**-**2**): 5 min; (**b**-**1**,**b**-**2**): 10 min; (**c**-**1**,**c**-**2**): 30 min; and (**d**-**1**,**d**-**2**): 30 min.

**Figure 4:**
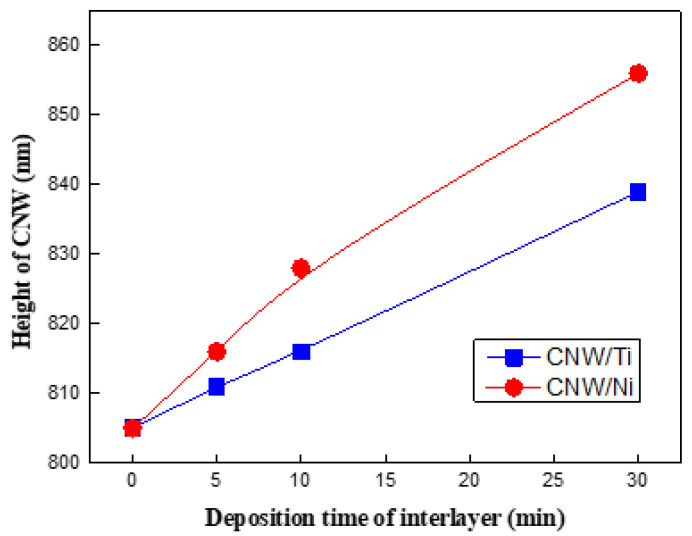
The height of the grown CNWs depends on the interlayers and deposition time.

Figure 3a–c shows the cross-sectional FE-SEM images of the CNWs after the interlayer was inserted between the CNW and the glass substrate. Figure 3d shows cross-sectional SEM images of the CNWs at 30 min of deposition time, depending on the different interlayers. It is difficult to confirm the thickness of the interlayers by FE-SEM measurement because the thickness of the coated film is very thin when the deposition period is less than 30 min. Based on these results, Figure 4 shows that the lengths of the CNWs vary depending on the deposition time of the interlayers. It was found that there were similar trends for both types of used interlayers; the CNWs became significantly longer as the deposition time was extended from 5 to 30 min, and all samples synthesized on the interlayer showed a higher height than the originally grown CNW due to the influence of the interlayer thickness. These graphs confirm that the height of CNWs grown on the Ni interlayer increased from 805 nm to 856 nm, and the height of CNWs grown on the Ti interlayer increased from 805 nm to 839 nm. These also indicate that the Ni and Ti interlayers not only improved the adhesion of CNWs to the glass substrate but also promoted their height. However, due to the effect of different deposition rates, the height of CNW produced on the Ti-interlayer-coated glass substrates was lower than on the Ni-interlayer-coated glass substrates for the same condition. In particular, the height difference of the CNWs according to deposition time was greatest at 30 min.

**Figure 5:**
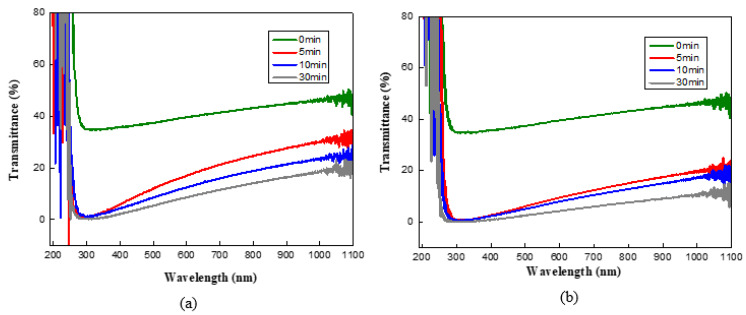

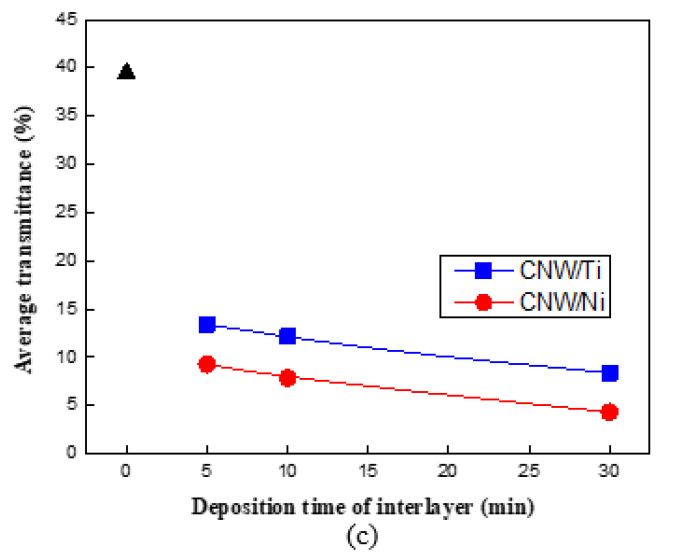
Light transmittance of the CNWs according to the different interlayers and deposition time: (**a**) CNW/Ti/glass, (**b**) CNW/Ni/glass, and (**c**) average transmittance of the 400–800 nm wavelength.

Figure 5a,b shows the transmittance waveforms depending on the wavelength, while Figure 5c presents the average transmittance of the 400–800 nm wavelength. The average light transmittance of CNWs directly on glass substrates is the highest at roughly 39.4%. Although the height of the CNW changed insignificantly after the interlayers were inserted at 5 min deposition time, the average transmittance dramatically reduced to about 16.10% for the Ti interlayer and 9.2% for the Ni interlayer. This seems to indicate that the interlayers have a strong light absorption capability. In addition, Figure 5c shows that the average transmittance of CNWs synthesized on both Ni and Ti interlayers decreased when the deposition time increased. Based on the SEM analysis, the CNW synthesized on the Ni interlayer was higher and denser than the CNW synthesized on the Ti interlayer, thereby resulting in a lower average transmittance.

**Figure 6:**
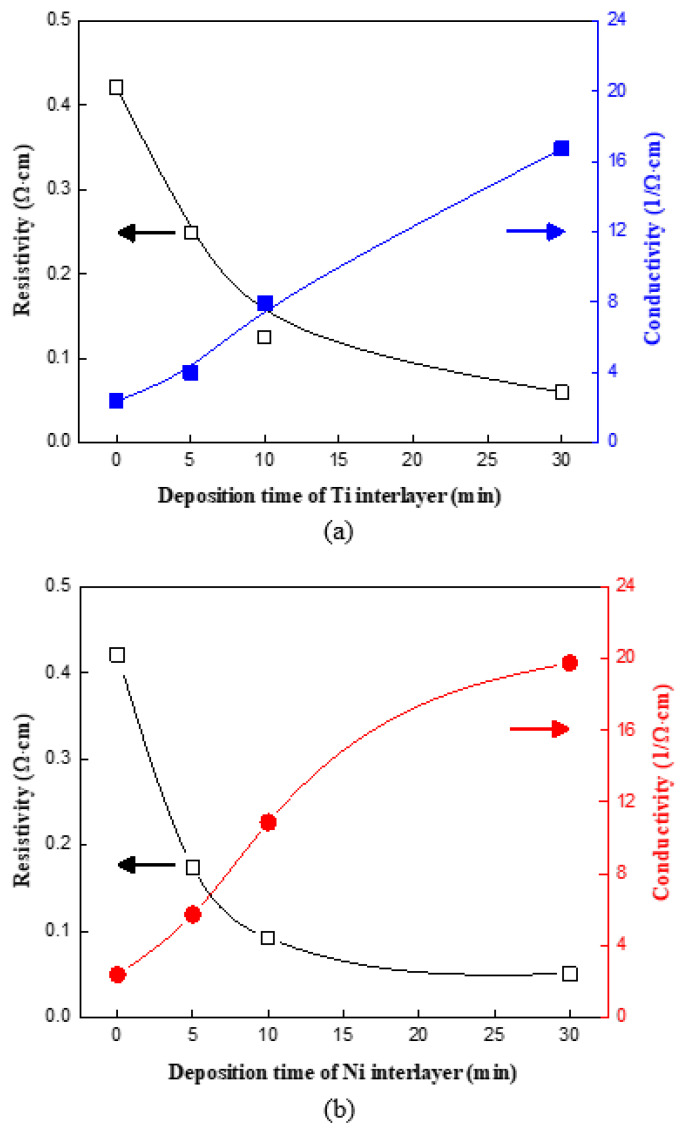
The resistivity and conductivity of the CNWs synthesized on an interlayer: (**a**) CNW/Ti/glass substrate and (**b**) CNW/Ni/glass substrate.

Figure 6 shows the graph in which the resistivity and the conductivity characteristics measured by using the Hall measurement are arranged. The resistivity graph shows that the resistance values of all the grown CNWs on the used interlayer appeared to be lower than those of the grown CNWs without an interlayer. Based on the SEM analysis, the CNWs that were synthesized on the interlayer-coated glass substrate were higher and far more densely grown than on the glass substrate. The resistivity reduced as the deposition time increased. The resistivity is lowest at 30 min of deposition time, roughly 0.06 Ω.cm for Ti interlayers and 0.05 Ω.cm for Ni interlayers. For both Ni and Ti interlayers, the maximum resistivity fell by 88.1% and 85.71%, respectively, when compared to the minimum value. The conductivity of the synthesized CNWs can be expressed as the reciprocal of resistivity. Furthermore, the graph shows that the Ni interlayer enhanced the conductivity of the CNW more effectively than the Ti interlayer under the same synthesis conditions. This is due to the fact that Ni has a higher electrical conductivity than Ti [18]. Another explanation is that Ti not only exhibits less deposition than Ni, but is also oxidized and transformed into TiO_2_ when it is exposed to the atmosphere at room temperature.

## 4. Conclusions

In this research, the effect of the interlayers according to deposition time was investigated in order to improve the use of CNWs. The CNWs were synthesized on either a Ni-interlayer-coated glass substrate or Ti-interlayer-coated glass substrate via microwave PECVD using a mixture of CH_4_ and H_2_ gases as the reaction gases. The FE-SEM analysis confirmed that the growth height and shape and growth density were different between CNWs grown directly on the glass substrate and CNWs grown on the interlayer. However, the adhesion of CNWs and their height were improved. Moreover, compared to the Ti interlayer, CNWs grown on the Ni interlayer were higher and had more density. According to the light transmittance analysis, the average transmittance dropped as the deposition time rose for both types. The resistivity in the Hall measurements appeared to have also decreased more significantly in all the synthesized CNWs than in the as-deposited CNW. Furthermore, the most outstanding resistivity and conductivity were observed at the deposition time of 30 min. The CNW’s electrical characteristics improved when the interlayers were inserted.

Based on this study, the CNWs grown on nickel and titanium interlayers not only presented with excellent adhesion, but also improved electrical characteristics, especially when the Ni interlayers were used. Therefore, carbon-based materials synthesized on either a nickel or titanium interlayer are likely to be used in a wide range of applications through additional studies in the future.

## Figures and Tables

**Table 1:** Deposition Conditions of the Interlayers via the RF Magnetron Sputtering System.

Parameters	Conditions
Substrate lass	Below 1 × 10^−6^ Torr
Working pressure	3 × 10^−3^ Torr
RF power	50 W
Deposition temperature	Room temperature
Target	Ni, Ti
Sputtering gas	Ar, 40 sccm
Rotation speed	1700 rph
Deposition time	5 min, 10 min, and 30 min

**Table 2:** Growth Conditions of the CNWs.

Parameters	Conditions
Substrate	Interlayer-coated glass substrates
Base pressure	Below 1 × 10^−5^ Torr
Working pressure	1.1 × 10^−2^ Torr
Microware power	1200 W
Substrate temperature	500 °C
Reaction gas	CH_4_: 20 sccm
Reaction gas	H_2_: 40 sccm
Growth time	5 min

## Data Availability

Not applicable.
